# Lymphatic Chyle Duct Injury and Identification During Laparoscopic Sleeve Gastrectomy Preventing Postoperative Chylous Ascites

**DOI:** 10.1007/s11695-024-07215-3

**Published:** 2024-04-08

**Authors:** Ahmed Abokhozima, Mohamed H. Zidan, Ahmed Abo Elmagd, Mohammed Alokl, Hashem Altabbaa, Mohamed Al Sayed, Aliaa Selim

**Affiliations:** 1 Alexandria University, El-Shatby, 22 El-Guish Road, Alexandria, 21526 Alexandria Egypt; 2 Alexandria Main University Hospital, Al Mothaf, Al Mesallah Sharq, Al Attarin, Alexandria, 5372066 Egypt; 3 Ekbal Hospital, 10 Hassan Amin Street, Alexandria, Egypt

**Keywords:** Chyle duct injury, Chylous ascites, Lymphatic duct injury, Laparoscopic sleeve gastrectomy

## Abstract

We present a case of intraoperative detection of an iatrogenic chyle duct injury during laparoscopic sleeve gastrectomy. The chyle duct injury was identified and managed by ligature, preventing postoperative chylous ascites.

**Supplementary Information:**

The online version contains supplementary material available at 10.1007/s11695-024-07215-3.

## Introduction

Chylous ascites (CA) or chyloperitoneum (ChP) is defined as the presence of intra-peritoneal triglyceride-rich chyle, due to chyle duct injuries (CDI) [1]. Chylous ascites is an uncommon clinical presentation first described in 1912, as an accumulation of chyle from intestinal or thoracic lymph [2]. There is no recent data regarding the incidence of chylous ascites, and the last dated study in 1984 indicated an incidence as low as 1 per 20,000 hospital admissions [3, 4]. The most common causes of CDI are atraumatic causes [1] and lymphatic anomalies [5].

CDI following LSG typically occurs due to extensive dissection or thermal injuries to one of the lymphatic duct tributaries of the cisterna chyli near the hiatus [6]. While CDI during bariatric surgery has been documented, not all surgeons are proficient in recognizing this rare complication [7]. In this video article, we are presenting a case of CDI during LSG that was identified and managed intraoperatively, to both raise the knowledge of this rare complication and highlight the possibility of its intraoperative management, avoiding a more complicated postoperative course.

### Case Presentation

A 25-year-old female patient, presented to our clinic complaining of class III obesity with a BMI of 45 kg/m^2^, with no past medical or surgical history. The patient’s pre-operative workup laboratory and ultrasonography were unremarkable. The patient was planned and prepared for LSG.

**Fig. 1:**
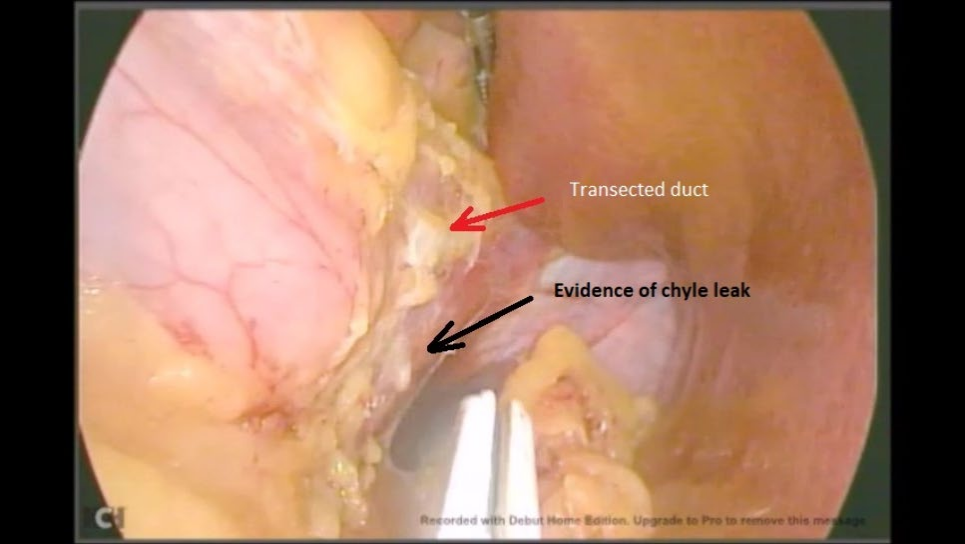
Evidence of chyle leak during dissection at the hiatus

**Fig. 2:**
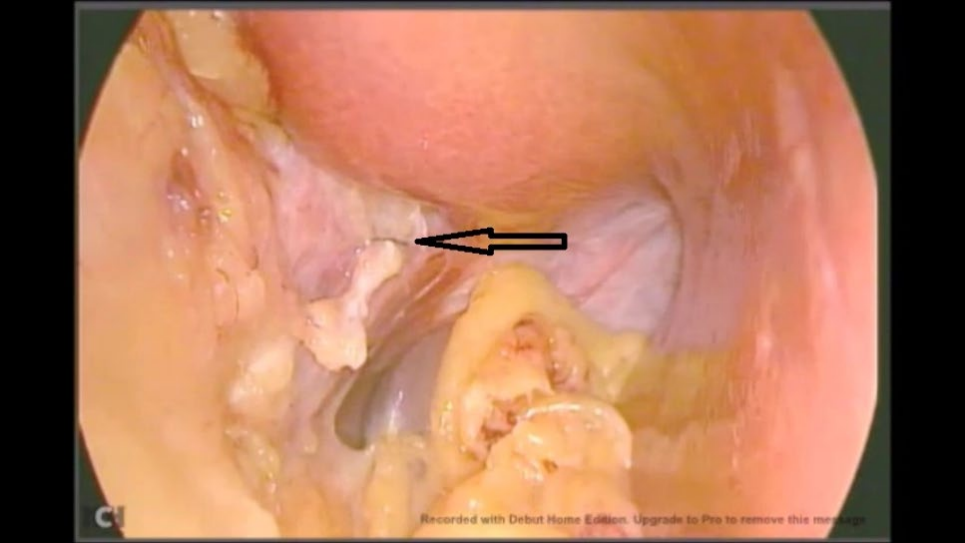
Clipping of the duct at the site of injury

**Fig. 3:**
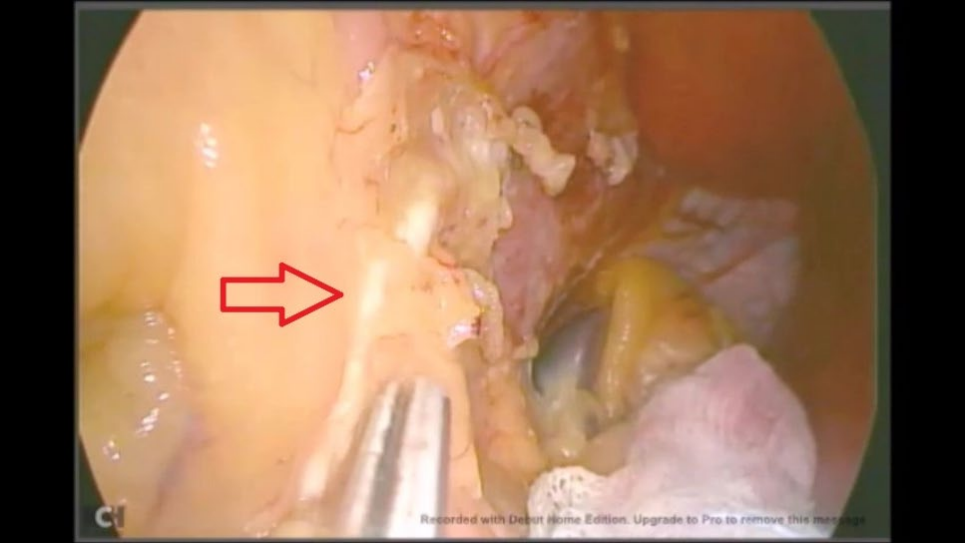
Identification of the duct proximally

**Fig. 4:**
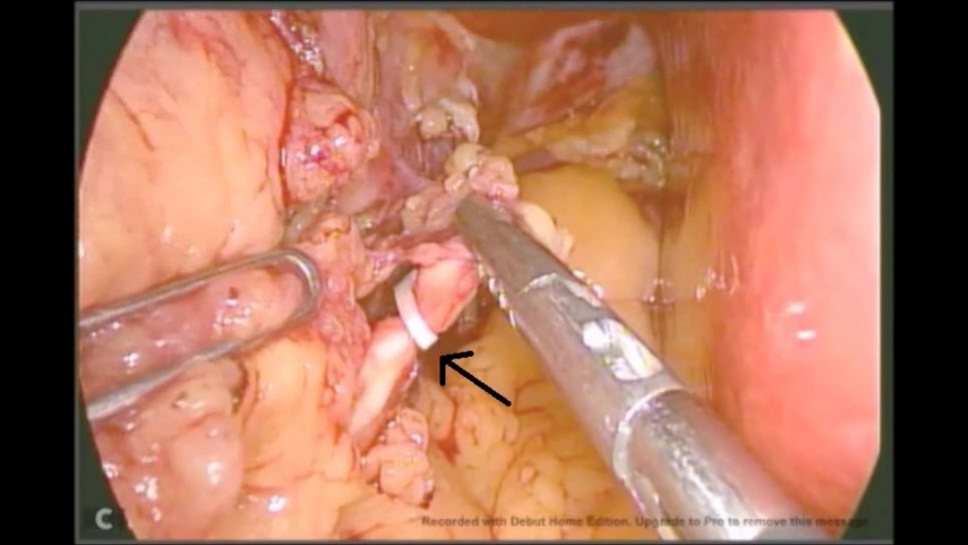
Dissection of the proximal duct, and ligature using a vascular clip

Unfortunately, due to a technical issue on the day of the operation, we were forced to use an older video system. Intraoperatively, there were no surgical difficulties during dissection encountered up until the dissection of the hiatus. A white structure was noted during the anterior dissection near the hiatus, which was misinterpreted as the white line of the left crus. Dissection over the peritoneal covering of this structure inadvertently showed a chyle leak (Fig. 1). The duct was identified and clipped at the site of leakage (Fig. 2) and stapling of the greater curvature and fundus was completed. After stapling, the duct was dissected caudally (Fig. 3) and ligated using a vascular clip (Fig. 4). An intraoperatively placed drain was left at the surgical bed, for follow-up postoperatively, and the excised stomach was extracted via the left mid-clavicular port, and the wounds were closed.

Postoperatively, the patient showed a good postoperative course, with no signs of fever, tachycardia, tenderness, or fluid spillage from the inserted drain. However, the patient was discharged on the first postoperative day on oral fluids, with the drain left until the first clinic visit 5 days later. The patient did not need any octreotide or any specific medications. The drain was removed on the 5th postoperative day at the clinic, and the patient was well with no mentioned complications. On follow-up after 6 months, the patient showed an uneventful recovery with a remarkable estimated weight loss (EWL%) of 40% of their body weight.

## Discussion

Chylous duct injury (CDI) is a potential complication following upper abdominal surgeries such as bariatric surgeries, including laparoscopic sleeve gastrectomies (LSG) [6, 8] and hiatal surgeries [9]. However, the available information regarding CDI is limited to case studies and case series, leaving no clear guidelines for its management, particularly when encountered during or after bariatric surgeries.

We conducted a mini-review of the literature using the PubMed search engine to identify cases of Chylous ascites related to bariatric surgeries. We excluded cases due to internal hernias or non-bariatric cases. Out of the 5 articles included [6–8, 10, 11], we identified 6 cases, out of which 3 cases were due to iatrogenic CDI, 2 cases were possibly due to band erosion after laparoscopic adjustable gastric band (LAGB) placement, and one case with an unknown cause following sleeve gastrectomy. We included all 6 cases in Table 1, including the timing of detection and the management modality undertaken, and added our case for comparison. We have also added a PRISMA flow chart of our search process, with data analysis and results available in Supplementary File 2 for further reading.

**Table 1:** Summary of all cases identified with CDI after or during bariatric surgeries from our review of the literature

Published articles	Age	Gender	BMI	Surgery name	Time of diagnosis	Time of diagnosis from surgery	Diagnostic modality in the postoperative setting	CT scan	Lymphangiogram	Fluid analysis	TG Level	Type of injury	Laparoscopy Vs. laparotomy in treatment	Site of injury	Management technique
Nau p et al. (2011) [10]	62	F	N/A	LAGB	Postoperative	5 months	Laparotomy	Yes	No	Yes	N/A	Lymphatic duct/chyle duct injury possibly due to band erosion	Laparotomy	Retroesophageal space where the band was placed	Removal of the band and re-enforcement ligation of the retro esophageal tunnel of which the band traveled, and application of fibrin glue
Nau p et al. (2011) [10]	61	F	N/A	LAGB	Postoperative	N/A: “Shortly after placement of LAGB”	Laparotomy	Yes	No	No	N/A	Lymphatic duct/chyle duct injury possibly due to band erosion	Laparotomy	Retroesophageal space where the band was placed	Removal of the band and re-enforcement ligation of the retro esophageal tunnel of which the band traveled, and application of fibrin glue
Halawani HM et al. (2016) [7]	42	F	39	LSG	Intraoperative	Intraoperative	Intraoperative (*de visu*)	No	No	No	N/A	Iatrogenic lymphatic duct/chyle duct injury	Laparoscopy	Left crural pillar	Ligation of the lymphatic duct with 2–0 *Polypropylene* sutures
Khogeer A et al. (2020) [6]	38	F	27	LSG	Postoperative	10 days	Lymphangiogram	No	Yes	Yes	278 mg/dl	Iatrogenic lymphatic duct/chyle duct injury	Laparoscopy	A small tributary of the cisterna chyli, seen near the hiatal surface posterior to the stomach	After attempting conservative measures for 4 weeks, the patient underwent laparoscopic exploration. The leaking duct was ligated with non-absorbable sutures and wrapped with a thrombin patch
Bora Makal G et al. (2020) [8]	33	F	42.7	LSG	Postoperative	2 days	drain fluid analysis	Yes	No	Yes	391 mg/dl	Iatrogenic lymphatic duct/chyle duct injury	Conservative	Unknown	Conservative management
Buschel HB et al. (2023) [11]	65	M	N/A	LSG	Postoperative	9 months	Laparotomy	Yes	No	No	N/A	Unknown	Laparotomy	Unknown	Lavage and drainage followed by conservative management
Our case	25	F	45	LSG	Intraoperative	Intraoperative	Intraoperative (*de visu*)	No	No	No	N/A	Iatrogenic lymphatic duct/chyle duct injury	Laparoscopy	Left crural pillar	Ligation of the lymphatic duct with a vascular clip

*F* female, *M* male, *N/A* not available, *LAGB* laparoscopic adjustable gastric band, *LSG* laparoscopic sleeve gastrectomy

Iatrogenic CDI can occur after any hiatal dissection and usually goes unnoticed. The normal anatomical location of the cisterna chyli and the thoracic duct behind the crura makes the identification of the lymphatic duct non-standardized; however, chyloperitoneum (ChP) has been reported after various hiatal surgeries, including laparoscopic Nissen fundoplication [12]. ChP has also been reported to occur after bariatric surgeries, mostly due to internal hernias, especially after RYGB. Sakran et al. [13] for instance had undergone a systematic review of the literature and identified 38 patients from 22 case reports and one cohort study [14] with postoperative ChP. In his study, Sakran et al. concluded that most of the cases were due to internal hernias after RYGB, and rarely due to iatrogenic CDI.

In our review, we have noted that out of the 6 cases included, 5 cases were identified in the postoperative setting, and only one case was identified Intraoperatively, making our case the second recorded case of intraoperative detection. We also noted that CDI was identified in the setting of LSG in 4 cases, and after LAGB in 2 cases.

Although it is understandable that iatrogenic CDI after bariatric surgeries should be detected in the intraoperative or immediate postoperative settings, our review has revealed that the average diagnosis period was 72 days (minimum: 0–maximum: 270). We observed this long period of diagnosis in two cases after LAGB, which may be attributed to the time taken for the band to erode the chyle duct. However, in other cases, the reason behind the long period of diagnosis was not mentioned, but it is possible that it was due to a missed contained leak postoperatively, which might have become symptomatic later in the course. Our data is insufficient to prove this theory; therefore, further research is needed to evaluate other rare causes of ChP in the postoperative setting, other than iatrogenic CDI and internal hernias.

**Fig. 5:**
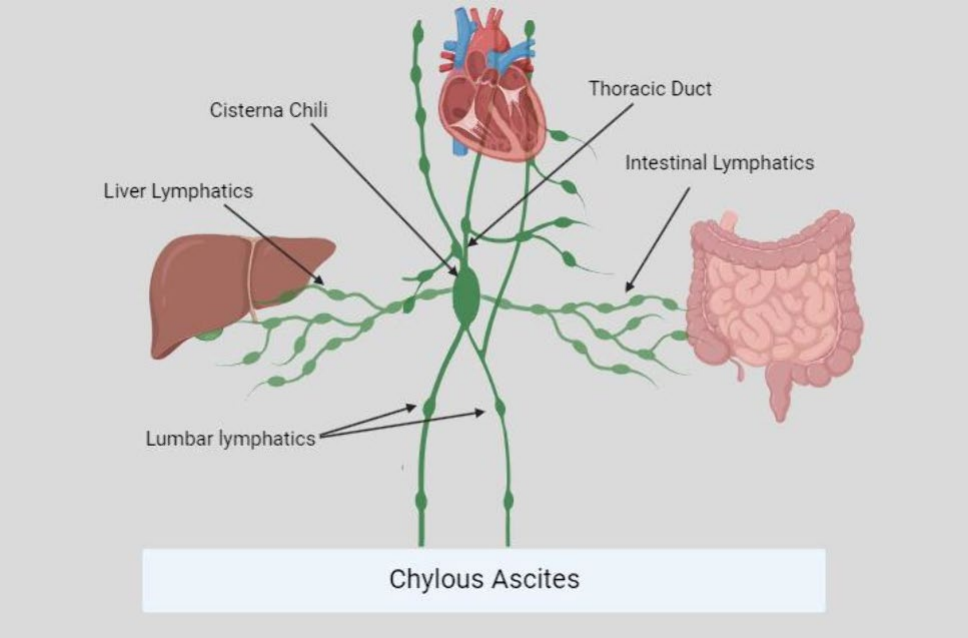
Chyle duct anatomy

Anatomically, intestinal, lumbar, and inferior intercostal lymphatics drain in the cisterna chyli at the level of L2, posterior to the crura. These, in turn, drain collectively into the thoracic duct and eventually terminate in the left internal jugular vein [15]; however, anatomical variations are common and are up to 50%, with the most common variation being a double duct system originating from the cisterna chyli at the diaphragmatic level, posterior or anterior to the crura [16]. Therefore, knowledge of the anatomical variations should be a prerequisite for surgeons performing hiatal, bariatric, and pancreatic surgeries (Fig. 5).

One of the risk factors of CDI is the usage of low-quality laparoscopy video systems. The use of old camera systems can hinder the surgeon in identifying rare anomalies and misinterpreting various anatomical abnormalities, which can increase the risk of overall morbidity. Advances in laparoscopic and robotic surgeries are associated with less intraoperative bleeding and complications [17]. These advancements in video systems should be implemented in all surgical centers, and surgeons should avoid the use of old sets even in presumably “easy cases.” This case highlights the need for high-quality video laparoscopic systems in all bariatric cases. It also teaches us a lesson to not underestimate the importance of advanced camera sets.

Knowledge of the anatomical variation possibility and intraoperative identification of ductal anomalies during bariatric surgeries can further decrease the rate of CDI, improving the patient outcome and avoiding unnecessary complications. Iatrogenic intraoperatively identified CDI can be treated by simple ligature or clipping of the duct. However, if the injury went unnoticed, CA would develop and might need further follow-up and management. Managing CA may entail drainage, conservative follow-up, pharmacological therapy such as octreotide, and surgical management [18].

CDI after LSG might confuse surgeons as to the color of the drain output, which may indicate either leakage or pancreatic injury. This would put the patients to more postoperative interventions such as computed tomography, lymphangiography, multiple ultrasound-guided aspirations, and re-operation resulting in increased morbidity [13].

## Conclusion

CDI and CA are rare complications of LSG; however, knowledge of such complications should be a prerequisite for all bariatric surgeons. Lymphatic duct injury can be avoided during LSG, through proper dissection, and identification of the hiatus, with a background knowledge of the possible anatomical variants of CD that could be anticipated. Undermining the use of advanced camera sets during bariatric surgeries might increase the risk of CDI. Management of intraoperative CDI can be treated by ligation of the duct. Therefore, we recommend that further studies should focus on CDI, its prevention, and evaluating its postoperative outcome.

### Supplementary Information

Below is the link to the electronic supplementary material.

Supplementary file1 (MP4 432042 KB)

Supplementary file2 (PDF 221 KB)

## Data Availability

All data generated in this article is available in Supplementary File 1.

## Abbreviations

CA: Chylous ascites
ChP: Chyloperitoneum
CDI: Chyle duct injury
LSG: Laparoscopic sleeve gastrectomy
RYGB: Roux-en-Y gastric bypass
BMI: Body mass index
LAGB: Laparoscopic adjustable gastric band
