# Supplementary material for: Lymphatic Chyle Duct Injury and Identification During Laparoscopic Sleeve Gastrectomy Preventing Postoperative Chylous Ascites
Source: Obes Surg. 2024 Apr 8;34(5):1995–2000. doi: 10.1007/s11695-024-07215-3 (PMC11031454; doi:10.1007/s11695-024-07215-3)
Supplement: Supplementary file 2 — Supplementary file2 (PDF 221 KB) [file 11695_2024_7215_MOESM2_ESM.pdf]

Supplementary materials

We have conducted a systematic review of literature identifying all cases of CDI during or after bariatric surgeries, and have excluded internal hernias. We have conducted a PRISMA flow chart depicting our search strategy in Figure 1S below. Our findings were tabulated in Table 1 in the main article. Some data were analyzed and are depicted below. These data aim to be available for further reading, or for researchers who are willing to build up on our data. The corresponding author is willing to share the data analysis for further research, and the data is available upon request.

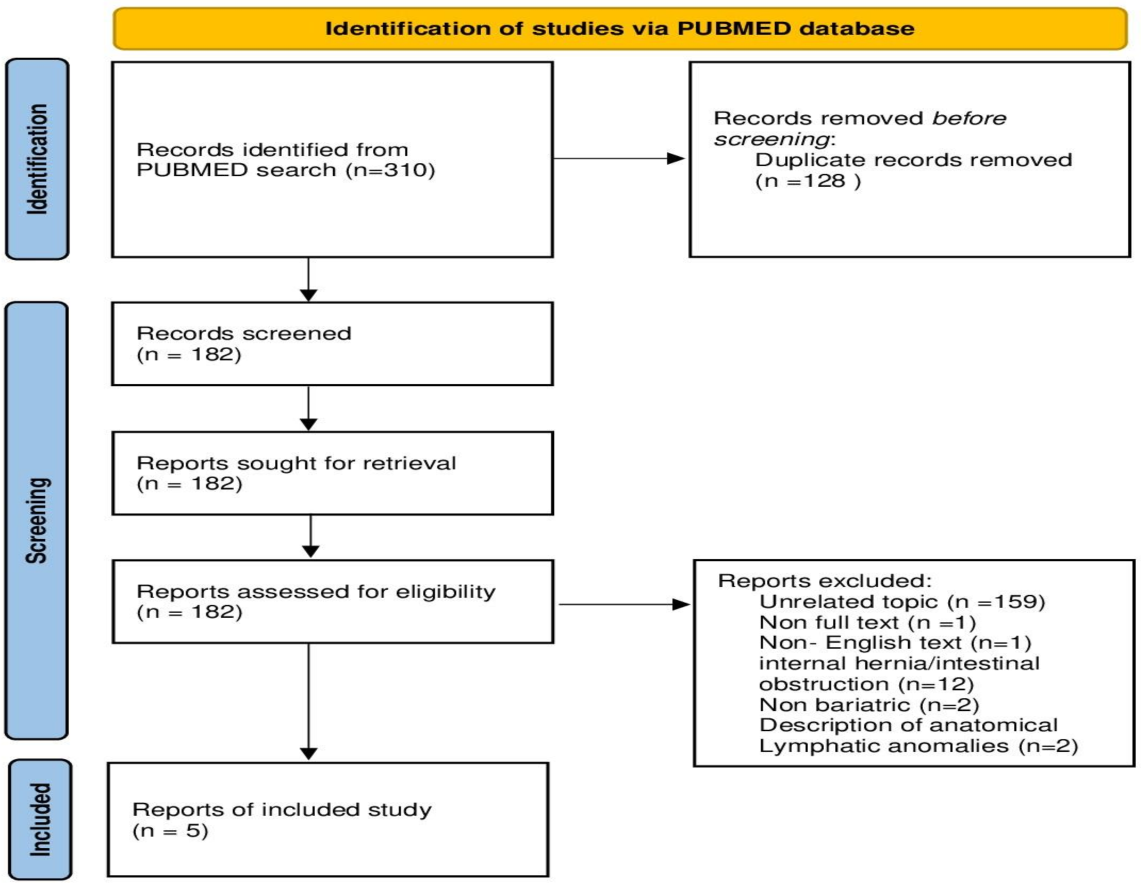

Figure 1: A PRISMA flow chart dictating our search strategy.

Table 1: Demographics identified

Descriptive Statistics

	N	Minimum	Maximum	Mean	Std. Deviation
Age	6	33	65	50.17	14.049
BMI	3	27	43	36.33	8.327
TG Level	2	110	391	250.50	198.697

Table 2: Sex identified from our study.

Sex

		Frequency	Percent	Valid Percent	Cumulative Percent
Valid	Male	1	16.7	16.7	16.7
	Female	5	83.3	83.3	100.0
	Total	6	100.0	100.0	

Table 3: Surgery where CDI was identified

Surgery Name

		Frequency	Percent	Valid Percent	Cumulative Percent
Valid	LAGB	2	33.3	33.3	33.3
	sleeve gastrectomy	4	66.7	66.7	100.0
	Total	6	100.0	100.0	

Table 4: Time of Diagnosis of CDI

Time of Diagnosis

		Frequency	Percent	Valid Percent	Cumulative Percent
Valid	Post-operative	5	83.3	83.3	83.3
	intra-operative	1	16.7	16.7	100.0
	Total	6	100.0	100.0	

Table 5: management modality undertaken

Laparoscopic Vs Laparotomy Vs Conservative management

		Frequency	Percent
Valid	Laparoscopy	2	33.3
	Laparotomy	3	50
	Conservative treatment	1	16.7
	Total	6	100.0

Table 6: Diagnosing modalities used

Diagnosing modalities used

		Frequency	Percent
Valid	CT	4	66.7
	intraoperative	1	16.7
	lymphangiogram	1	16.7
	drain fluid analysis	3	50
	Total	6	100.0
